# Commentary: Causal Effects in Mediation Modeling: An Introduction with Applications to Latent Variables

**DOI:** 10.3389/fpsyg.2017.00151

**Published:** 2017-02-09

**Authors:** Emil N. Coman, Felix Thoemmes, Judith Fifield

**Affiliations:** Health Disparities Institute, UConn HealthFarmington, CT, USA

**Keywords:** mediation, causal mediation, potential outcomes, causal inference, counterfactuals

Causal mediation^1^ is an increasingly popular analysis, as recently described by Muthén and Asparouhov (2015, M&A)^2^. We suggest a simplified notation for causal mediation effects, **i**_T_/*i*_*P* = *BK*_ and **d**_T_/*d*_*P*_, provide a graphical view of potential outcomes (PO) and expand the M&A approach by using VanderWeele's (2014) mediation decomposition.

An intuitive way to label and see causal in/direct effects is to directly display POs, as in Figure 1 below. POs are values that could be observed, but have not been realized (yet). They reveal themselves partially once nature or researchers assign people to specific experimental conditions, or when people make choices. POs are useful in defining causal total effects (TE), as differences between the same individual's (*i*) two POs, Y_*i*__1_ – Y_*i*__0_, had the person been treated (subscript 1), and alternatively (but *simultaneously*) not treated (0); evidently, in our reality one of these has to be “contrary-to-fact” (CF).

**Figure 1:**
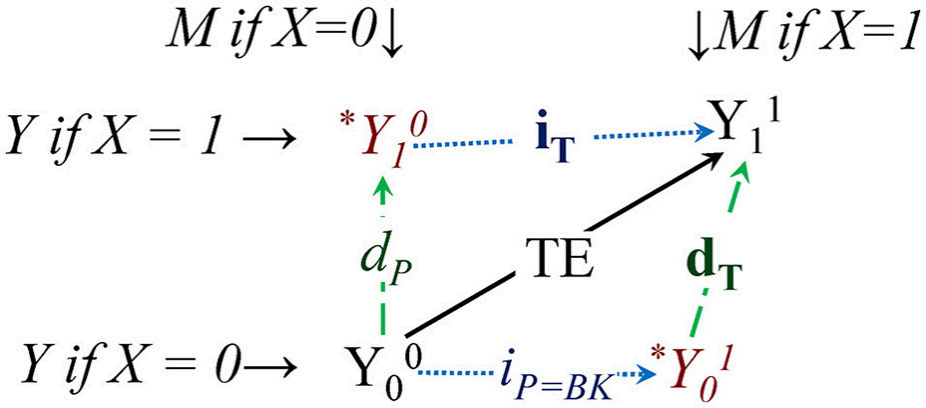
**Causal mediation represented with potential outcomes (POs)**. ^*^$Y_{0}^{1}$ is the PO of Y is X was set to 0 (subscript) but M took the value it would attain if X was instead set to 1 (superscript); ^*^ means that PO is unobservable/contrary-to-fact (CF). The upper level is the potential world if treated (X = 1), the bottom if not treated (X = 0); *i*_*P* = *BK*_/**i**_T_ and *d*_*P*_/**d**_T_ are pure/total indirect and direct effects (BK comes from the “classic” Baron-Kenny). The total effects (**i**_T_ and **d**_T_, in bold) are shown as longer than their pure counterparts, *i*_*P* = *BK*_ and *d*_*P*_ (in *italics*), both by exactly INTMed, the mediated interaction. The arrows from lower to upper PO worlds are the two causal direct effects *d*_*P*_ and **d**_T_, those from the left-side POs to the right-side POs capture the indirect effects *i*_*P* = *BK*_ and **i**_T_, while the diagonal up and to right the total effect TE. TE can be decomposed then as *d*_*P*_ + **i**_T_, or *i*_*P* = *BK*_ + **d**_T_.

The indirect effect of X on Y through a mediator M is the part of the total effect that “flows through” M, or the contribution of the path X->M->Y to the observed association between X and Y, which is an open path because causal association flows through it (Elwert, 2013). The key problem in intuitively grasping causal in/direct effects is the “nesting” of the POs due to the double role of the mediator as a cause *and* an effect^3^: the PO “Y if X was set to x,” or Y_x_, can be combined with “Y if M was set to m,” or Y^m^ (we suggest using a superscript for scenarios involving M). So ^*^${\text{Y}}_{1}^{\text{M}0}$, for example, labeled Y(1, M(0)) in M&A, is the PO of the outcome Y if a person was treated (_1_), but his/her mediator took on the value had s/he would belonged to the opposite (control) condition (^M0^). This PO is clearly contrary-to-fact (CF), never observable, a “cross-worlds” quantity (Lok, 2016), hence our ^*^ sign. ${\text{Y}}_{0}^{0}$ and ${\text{Y}}_{1}^{1}$ are in principle realizable, only one of them at a time for the same person, however.

The four key POs involved in understanding causal in/direct effects are shown in Figure 1. The total effect is decomposable into direct and indirect causal effects, possibly in two ways, through one of two fully contrary-to-fact POs: ^*^${\text{Y}}_{1}^{0}$ or ^*^${\text{Y}}_{0}^{1}$.

Both decompositions of TE can be obtained by adding and subtracting a fully CF intermediary term; e.g., through ^*^${\text{Y}}_{1}^{0}$:

(1) $$\text{TE}={\text{Y}}_{1}^{1}-{\text{Y}}_{0}^{0}=({\text{Y}}_{1}^{1}{-}^{*}\text{​}{\text{Y}}_{1}^{0})+{(}^{*}{\text{Y}}_{1}^{0}-{\text{Y}}_{0}^{0})=i_{T}+{\text{d}}_{\text{P}}$$

Intuitively, one can see that the two vertical arrows are direct effects, because they capture the “change” in Y (in the PO world), marked by subscript/superscript changes: when “changing” only X, i.e., while (un-naturally) holding the mediator at a “constant” PO-value. The causal pure direct effect *d*_*P*_ is often referred to as natural (or pure natural direct effect, PNDE, in M&A), because the mediator takes on the same value under the control condition, which would be the “natural” course of action without any change in nature.

Similarly, the two horizontal arrows are indirect effects, because they are the result of “changing” only M, while keeping X constant (at 0, or 1)^4^. The “upper” indirect effect is called also natural, but is in fact a *total* indirect effect (total natural indirect effect, TNIE, in M&A); it is *total* because it is a sum, of its *pure* kind, which we label *i*_*P* = *BK*_, and an interacted mediation component, see Equation (3) below; here X is kept “unchanged” at the treated level (1), yet the mediator “changes” its (potential) value, from its natural (control) value to the value “if treated.”

We suggest to label the *pure* indirect effect *i*_*P* = *BK*_, because its estimate for continuous M and Y matches the classic no interaction and no confounder Baron and Kenny (1986) indirect effect “a · b” (see Equation 8 in M&A, when an interaction X-by-M is specified).

The relation between the key causal effects **d**_T_ and *d*_*P*_ and **i**_T_ and *i*_*P* = *BK*_ has been revealed by VanderWeele's decomposition (VanderWeele, 2014), hence the *total* labels we proposed:

(2) $$d_{\text{T}}=d_{P}+{\text{INT}}_{\text{Med}}\text{ and }i_{\text{T}}=i_{P=BK}+{\text{INT}}_{\text{Med}},$$

where INT_Med_ is the mediated interaction component^5^, which is the product of the interaction estimate and the X->M linear effect, $\beta_{{\text{X}}^{*}M}$ · a, labeled γ_1_· β_3_ in M&A, see their Equations (5) and (9); INTMed is non-zero when X impacts M, and X and M interact in how they impact Y.

Because the Mplus software code in M&A for computing causal in/direct effects did not estimate the effects proposed by VanderWeele's “decomposition” (mediated interaction, controlled direct effect, proportion attributable to interaction, and portion eliminated), we expand the Mplus code for continuous M and Y to estimate them (see the online appendix at https://bit.ly/pos_frontiers); we present an expanded VanderWeele SAS code too, which estimates the Mplus additional effects: pure direct, total indirect and total direct.

To illustrate, we estimated effects from a weight-loss randomized intervention data (SisterTalk Hartford, Burleson et al., 2008; de-identified data for replication available in appendix), which was meant to improve food habits and consequently reduce BMI in African-American women; effects are shown in Equation (3) (following VanderWeele's Figure 4, 2013, which is an expanded online version of the published (VanderWeele, 2014); ^*^ signals statistically significant at *p* < 0.05, NS signifies non-significant):

(3) $$\begin{array}{ccc} \begin{array}{l} \;TE\\ -0.663^{*} \end{array} & = & \begin{array}{l} \begin{array}{cc} \overset{d_{P}=-0.495^{*}(75\%)}{\overset{︷}{\;{\;}^{\text{m}}CDE\;+{\;}^{\text{m}}INT_{\text{Ref}}\;}} & +\overset{{\text{i}}_{\text{T}}=-0.168^{*}(25\%)}{\overset{︷}{INT_{\text{Med}}\;+BK\;}} \end{array}\\ \begin{array}{cc} \underset{{\text{ d}}_{\text{T}}=-0.474^{*}(71\%)}{\underset{︸}{-0.507^{*}(76\%)\;0.012^{\text{NS}}(-2\%)\;0.021^{\text{NS}}(-3\%)}} & \underset{\;i_{P=BK}=-0.189^{*}(29\%)\;}{\underset{︸}{\;-0.189^{*}(29\%)}} \end{array} \end{array} \end{array}$$

where *INT*_*Med*_ is the mediated interaction, BK is the “Baron and Kenny” causal indirect effect, ^m^CDE is the controlled direct effect, ^m^INT_Ref_ reference interaction, with superscript *m* signaling that those effects depend on what value *m* the analyst decided to estimate them at.

The total effect TE was −0.66 BMI units (approx. −3.9 lbs. for an average 64 inch woman). The mediated interaction effect *INT*_*Med*_ is about 3% of the TE, and statistically non-significant, hence statistically **i**_T_ $\overset{stat.}{=}\;$ *i*_*P* = *BK*_ and **d**_T_ $\overset{stat.}{=}$ *d*_*P*_ (“stat.” signals statistical, not mathematical, equality), so one can report the classic i_BK_^6^: the weight loss achieved through improving one's food habits is about 25% of the total effect, while the residual direct effect is about 75% of it.

While POs are central to “causal” mediation, visually “seeing” them is challenging, yet, when achieved, it helps uncover the mechanics behind causal direct and indirect effect estimation. Intuitive graphical displays could aid in visualizing some assumptions, many of which refer to relations between POs, and not their observed cousins (e.g., ignorability, or unconfoundedness, Imai et al., 2010); such assumptions ensure identifiability of in/direct causal effects.

We hope that the simplified notation and a visual display of how causal in/direct effects emerge from a mix of the POs of the mediator and the final outcome can contribute to a more intuitive understanding and reporting of causal mediation, as presented in the seminal paper we commented on. The notational bridge and cross-pollination of software syntaxes we suggested should facilitate such an improved understanding.

## Author contributions

ENC has developed the idea, FT has verified the claims, expanded, and revised the manuscript extensively, JF has worked on the theoretical and design portion of the original study and has revised and edited the manuscript.

## Funding

The Sistertalk Hartford project was funded by the Patrick and Catherine Weldon Donaghue Medical Research Foundation.

### Conflict of interest statement

The authors declare that the research was conducted in the absence of any commercial or financial relationships that could be construed as a potential conflict of interest.

## Footnotes

^1^The label “causal” mediation reflects more than the expansion of the original Baron and Kenny model to allow for X-by-M interaction, and does not suddenly make any three-variable model causal in the profound sense. Causal mediation relies on meeting other assumptions, like the no-confounder assumption of M and Y, and would require causal investigations like those afforded by Direct Acyclic Graphs (DAGs, Greenland et al., 1999).

^2^Other dominant causal mediation “schools” are led by the Imai (Imai et al., 2010) and Pearl (Pearl, 2001) teams, first centered on R and Stata implementations, the latter more theoretical and non-parametrical. They differ also in terms of formulating the assumptions for identification of the causal in/direct effects.

^3^Pearl (2013) calls them nested counterfactuals; the key insight Sewall Wright foresaw when proposing the path analytic method may have been that the change in Y in relation to the change in X (the slope δY/δX), traced on the path through an intermediary M, is linked to the slopes δM/δX and δY/δM following the composite function chain rule of derivatives: δy/δx = δy/δm · δm/δx, which mirrors the Baron and Kenny i = a · b. Adding the contributions of all such X-to-Y open paths yields the model predicted association between X and Y (see the “tracing rule,” Loehlin, 2004).

^4^The fact that there is possibly more than one indirect (and hence direct) effect to estimate follows from the interaction of X and M in causing Y, which makes the effect of M on Y vary with X (or the effect of X on Y vary with M).

^5^The INTMed key component is defined in VanderWeele (2013) as (${\text{Y}}_{1}^{1}$ − ^*^${\text{Y}}_{1}^{0}$ − ^*^${\text{Y}}_{0}^{1}$ + ${\text{Y}}_{0}^{0}$) (M_1_ – M_0_) which for continuous M and Y becomes either **i**_T_-*i*_*P* = *BK*_ or **d**_T_-*d*_*P*_.

^6^While we label the pure indirect effect *i*_*P* = *BK*_, as being the Baron and Kenny classic indirect effect, a · b, its estimate in the “causal” specification, with the X-by-M interaction term included, will not coincide of course with the estimate from the simpler model without interaction; in our case the classic BK estimate was i_BK_ = −0.179 (*SE* = 0.054), *p* < 0.001, while *i*_*P* = *BK*_ was −0.189 (*SE* = 0.069), *p* = 0.006.
